# Evolution and Influencing Mechanisms of the Yili Loess Mechanical Properties under Combined Wetting-Drying and Freeze-Thaw Cycling

**DOI:** 10.3390/ma16134727

**Published:** 2023-06-30

**Authors:** Yongliang Zhang, Zizhao Zhang, Wanhong Hu, Yanyang Zhang

**Affiliations:** 1 School of Geological and Mining Engineering, Xinjiang University, Urumqi 830017, China; zyl15193288192@163.com (Y.Z.);; 2 State Key Laboratory for Geomechanics and Deep Underground Engineering, Xinjiang University, Urumqi 830017, China

**Keywords:** WD-FT cycles, loess, shear strength index, microstructure, gray correlation

## Abstract

Landslides frequently occur in the loess-rich Yili region of Xinjiang, China, due to the combined effects of wetting-drying and freeze-thaw (WD-FT) cycles, which cause changes in the soil/loess internal structure and shear strength. This paper explores the combined effect of WD-FT cycles on the shear strength evolution of Yili loess through cyclic and triaxial shear tests. The micromechanism of the effect of WD-FT cycles on the loess properties is studied through scanning electron microscopy tests. Finally, the gray correlation analysis method assesses the correlation between relevant macro and micro parameters. The results show that: (1) With the increase in WD-FT cycles, the cohesion of loess decreases first and then gradually stabilizes, while the internal friction angle first grows and then drops before stabilizing. This indicates that the WD-FT cycles cause different degrees of decline in the soil’s internal friction angle and cohesion. (2) As the number of WD-FT cycles increases, the average abundance and directional probability entropy fluctuate slightly, gradually decreasing and stabilizing. In contrast, the particle size dimensionality gradually decreases and stabilizes, and the pore area ratio first increases and then gradually stabilizes. (3) Six microstructural parameters (average diameter, average abundance, particle size dimensionality, directional probability entropy, particle roundness, and pore area) are selected for correlation analysis with the shear strength index of loess. The results show that the particle size dimensionality closely correlates with macroscopic internal friction angle under coupled cycling, while the pore area closely correlates with macroscopic cohesion. These findings are instrumental in preventing and controlling loess landslides caused by WD-FT cycles in the Yili region of Xinjiang, China, and similar loess-rich regions.

## 1. Introduction

Landslides can be triggered by wetting-drying and freeze-thaw cycles, which deteriorate the soil’s internal structure and shear strength [1,2]. Freeze-thaw (FT) cycling implies the freezing and thawing of water in soil associated with the respective temperature changes in the winter months. Wetting-drying (WD) cycles are defined as alternating periods of wetting, saturation, and drying of soil or geomaterials, which are simulated in laboratory conditions by submerging the geomaterial under study in water until saturation and air-drying to the initial moisture content of about 20%. According to numerous reports, after repeated FT and WD cycles, unsaturated loess’s cohesion and internal friction angle drop exponentially with the number of cycles [3,4,5,6,7], while the soil shear strength decreases significantly in the early stages of WD-FT coupled cycling [8,9,10].

With the continuous development of science and technology, the soil/loess microstructure and its evolution have been explored by state-of-the-art techniques [11]. Several studies revealed that, due to high porosity and weak loess cementation, the soil aggregates underwent repeated splitting and aggregation during the WD-FT coupled cycling, weakening the interparticle bonding force and causing the soil particles to sink under gravity. After rearrangement, the pores in the soil increased, and the particle size, spacing, and pore size became more uniform [12,13,14].

This paper analyzes the correlation between loess’s shear strength and microstructure using the gray correlation method based on the Gray System Theory (GST) proposed in 1982 by Deng [15]. This method allows one to measure the degree of similarity between two systems using two indices: gray relational coefficient (GRC) and gray relational grade (GRG). The larger the value of GRC, the higher the GRG, which indicates the higher the similarity of the two systems or elements, and the smaller the value, the lower the similarity. Since 1982, this approach has been widely used in various fields. Problems in special areas where unknown factors exist can be solved, and it is widely used in disciplines such as agriculture, geology, and meteorology. Many researchers have successfully applied it to correlate the macroscopic properties and microstructure parameters of loess [16,17,18,19]. However, such analyses mainly focused on the mechanical strength of intact loess [20,21], while the correlation between soil’s shear strength index and its microstructural parameters under WD-FT coupled cycling has received much less attention. Therefore, studying the changes and mechanisms of the mechanical properties of loess under WD-FT coupled cycling is vital for a comprehensive understanding of the characteristics of loess in seasonally frozen soil areas.

The Yili Valley is one of the important distribution areas of seasonal permafrost, and the widespread and frequent loess landslides seriously affect the production and life safety of people in the Yili region. The seriousness and complexity of the loess landslide hazard in the Yili Valley and the severity of the disaster caused by repeated WD-FT cyclic action have not received sufficient attention in today’s rapid construction process. Among the 380 landslides in the Yili Valley for which time records are available, 152 are caused by the coupling action of WD-FT, accounting for 40% of the total number of landslides [22]. This shows that WD-FT coupled cycling strongly impacts slope stability [23,24]. In contrast, however, few research results exist on loess’s WD-FT coupled cycling in the Yili region.

This study attempts to fill this knowledge gap by experimentally investigating the mechanical properties’ changes and mechanisms of loess samples taken in the Yili region under WD-FT coupled cycling and analyzing the respective changes in the loess structure from a microscopic perspective. To this end, the gray correlation analysis method is used to analyze the correlation between macro and micro parameters of loess in the Yili region of Xinjiang, as well as identify the main factors affecting the mechanical properties of loess in this area. The research results are expected to provide a theoretical basis for preventing and controlling loess landslides caused by WD-FT coupled cycling in the Yili region and other sites with similar geological conditions.

## 2. Experimental Setups

### 2.1. Sample Preparation

**Figure 1:**
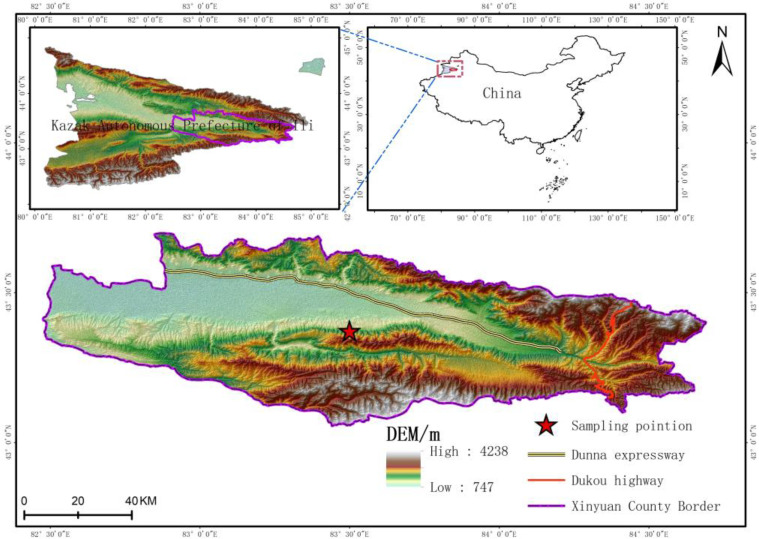
Diagram of the location of loess sampling in the study area.

For testing, samples of yellow soil (loess) were taken from the west side of Hayindesayi Gou near Alemale Town, Xinyuan County, Yili Prefecture (see Figure 1). The sampling depth for the test soil was 2 m, and the soil texture was uniform, gray-yellow to brown-yellow. After sampling, it was immediately wrapped in plastic to prevent moisture evaporation and fixed with tape. In total, about 30 kg of loess were collected. The basic physical properties of the soil were measured through indoor basic physical experiments (density test, moisture content test). According to the standard of geotechnical test methods, the blocky disturbed soil was spread out in a cool place for air drying. After the soil was air-dried and crushed with a wooden roller, it was sieved through a 2 mm sieve. The remolded sample was prepared by spraying water according to the natural moisture content of 20.51% using a spray device, thoroughly mixing and sealing it in a container, and placing it in a cool place to moisten for one day and night. The compaction method prepared the remolded sample with a maximum dry density of 1.86 g/cm^3^ (see Table 1).

### 2.2. Coupled WD-FT Cyclic Tests

**Figure 2:**
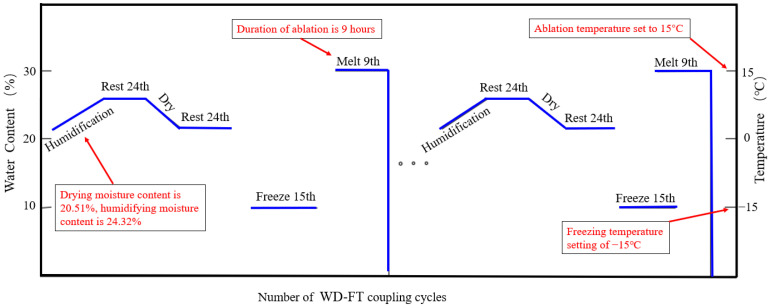
Diagram of WD-FT coupling cycle.

The prepared samples were sprayed with water to increase humidity, dried in an oven, and subjected to freeze-thaw cycles to simulate the effects of rainfall, evaporation, and freeze-thaw cycles in the Yili region. The natural moisture content of the samples was 20.51%, and the moisture content after experimental humidification was 24.32%. After humidification, the samples were placed in a moisture-retaining container and left to stand for 24 h to distribute the moisture evenly. After the WD cyclic test, the FT was immediately conducted using a JW-2000 series test box with constant humidity and temperature (±0.1 °C). According to the local weather conditions, the freezing temperature for the freeze-thaw cycle was set at −15 °C, with a freezing time of 15 h, and the thawing temperature was set at 15 °C, with a thawing time of 9 h (see Figure 2). The numbers of cycles set for the WD-FT coupled tests were 0, 1, 5, 10, 15, and 20.

### 2.3. Triaxial Shear Test

After undergoing a WT-FT coupled cycling, the sample’s preservation film, permeable stone, and PVC pipe were removed from its surface, and it was subjected to a triaxial shear test using a TFB-1 type unsaturated soil stress-strain controlled triaxial apparatus. This instrument mainly comprised a backpressure control system, a surrounding pressure control system, a pressure chamber, computer software control, and a data acquisition system.

This study mainly focused on the WD-FT coupled cycling effect on soil strength and microstructure, with consolidation influencing the effectiveness of this process to a certain extent. Therefore, the unconsolidated undrained (UU) rapid shear test was chosen for this experiment. According to the standard of geotechnical test methods, the shear strain rate was 0.6 mm/min (0.5%/min~1.0%/min), and the confining pressure values were set at 100, 200, and 300 kPa.

### 2.4. Scanning Electron Microscopy Examination

To obtain samples for microscopic changes after different numbers of WD-FT coupled cycling, the samples were cut into 1 cm^3^ pieces and examined via scanning electron microscopy (SEM) using an FEI Quanta 250 FEG field emission environmental scanning electron microscope. Before the SEM examination, the samples were dried in an oven, and their surface was coated with gold.

### 2.5. Gray Correlation Analysis

Gray correlation theory allows one to predict unknown information through partially known information, and its research system is incomplete in terms of information [25]. The macroscopic variation of loess is determined by some unknown microstructures, which belong to the category of gray system theory. Therefore, applying the gray system theory in this study to analyze the correlation of selected macro and micro parameters was scientifically substantiated. By comparing the gray correlation degree of each macro and micro parameter, the main and secondary relationships between each parameter were determined, and the relationships between macro and micro parameters were obtained.

## 3. Experimental Results and Analysis

### 3.1. Analysis of the Triaxial Shear Test Results

**Figure 3:**
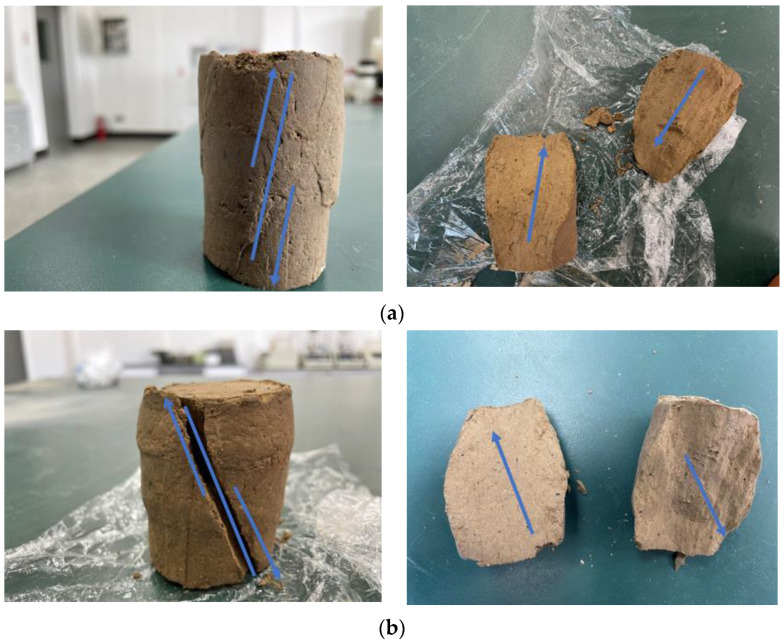
Shear damage pattern of specimens after triaxial compression test (**a**) First shear damage pattern; (**b**) Second shear damage pattern. (The blue arrows point to the direction of relative sliding of the soil shear damage).

After the indoor triaxial compression test of the sample, two different shear modes were realized. One shear surface was relatively intact with obvious displacement, as shown in Figure 3a; the other was more fragmented with a large protrusion at the bottom of the sample, as shown in Figure 3b. This difference was mainly related to the preparation method, the sample moisture content, and the number of WD-FT coupled cycles. The preparation method used in this experiment was the compaction method, which produced a somewhat uneven distribution of soil density. In addition, under repeated WD-FT coupled cycling, the soil’s moisture content always fluctuated, and the water-ice in the soil pores always underwent a conversion process. The moisture content of each part of the sample was unevenly distributed during the cycling process, and the moisture content was only approximately uniform at the end of the static process. The uneven moisture content led to an uneven distribution of pore water pressure, and the degree of deformation of the soil under shear stress varied, resulting in two different shear modes in the triaxial shear test.

### 3.2. Stress-Strain Curves

**Figure 4:**
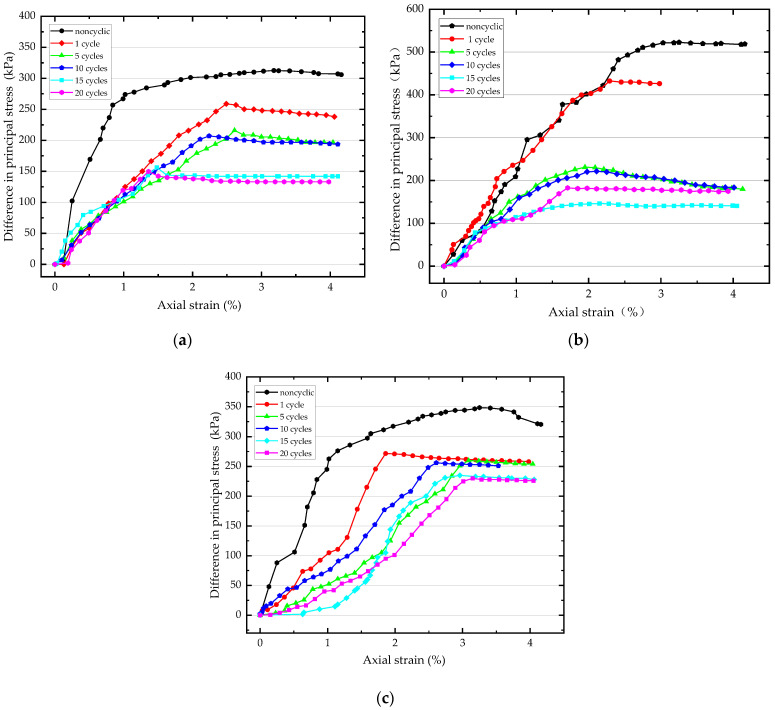
Stress–strain curve (**a**) Stress–strain curve after a different number of WD-FT coupling cycles at 100 kPa; (**b**) Stress–strain curve after a different number of WD-FT coupling cycles at 200 kPa; (**c**) Stress–strain curve after a different number of WD-FT coupling cycles at 300 kPa.

Six sets of triaxial shear tests were conducted on samples subjected to WD-FT coupled cycling. Each set consisted of three samples with confining pressures of 100, 200, and 300 kPa, respectively. Considering the actual state of the soil, a rapid shear test was chosen with a shear rate of 0.6 mm/min. The data from the experiments were processed to construct the principal/axial stress–strain curves after different numbers of WD-FT coupled cycles, as shown in Figure 4.

As seen from the figure, the variation trends of the principal stress difference of the sample under different confining pressures were generally consistent (an increase, followed by a decline, with some peaks). Compared with samples in the initial state, the samples experiencing WD-FT coupled cycling showed a decreasing trend in the principal stress difference. Only at a confining pressure of 100 kPa, the peak principal stress difference increased to a certain extent after one WD-FT cycle, mainly due to the change in the soil’s internal structure.

The analysis of test results revealed the following trends in the sample strength evolution after WD-FT coupled cycling: Under unconsolidated undrained (UU) rapid shear test conditions, the curve position after zero WD-FT cycles was the highest; it decreased with the number of WD-FT coupled cycles and then stabilized. The amplitude change was the largest in the early stages of coupled WD-FT cycling; the peak principal stress difference dropped with the increased number of coupled cycles. This indicates that the soil’s internal structure deteriorates with the number of WD-FT cycles, promoting its strength to change.

### 3.3. Shear Strength Inde

Based on the data obtained from indoor triaxial shear experiments, the Mohr-Coulomb theory (as shown in (1)) was used to analyze the experimental data, and the shear strength index of the Yili loess under different numbers of WD-FT coupled cycles was obtained. The calculation process was as follows [26]:

(1) τ=c+σtanφ

According to the Mohr–Coulomb stress circle:

(2) $$\sin\varphi =\frac{\frac{\sigma_{1}-\sigma_{3}}{2}}{c\cot\varphi +\frac{\sigma_{1}+\sigma_{3}}{2}}$$

After rearrangement, we obtain:

(3) $$\sigma_{1}=A+B\sigma_{3}\;$$

Among them:

(4) $$A=\frac{2C\cos\varphi }{1-\sin\varphi },\;B=\frac{1+\sin\varphi }{1-\sin\varphi }$$

**Figure 5:**
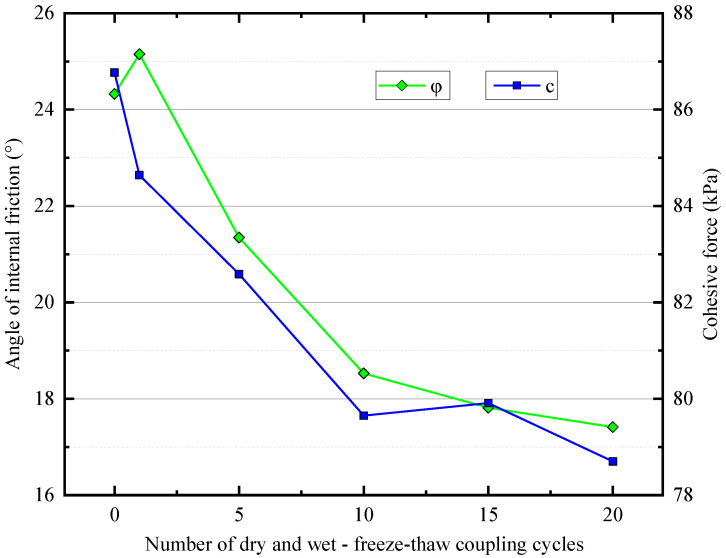
Variation of shear strength index with the number of couplings.

Figure 5 shows the relationship between the internal friction angle (φ) and cohesion (c), two shear strength indicators of the Yili loess, and the number of WD-FT cycles. Under the undrained unconsolidated (UU) conditions, compared with the samples with zero WD-FT cycles, the cohesion of the loess decreased first. It then gradually stabilized with the number of WD-FT cycles. At the same time, the friction angle increased first, then decreased, and finally gradually stabilized. This indicates that the WD-FT coupled cycling impacted the soil’s cohesion and friction angle, leading to the soil’s shear strength changes.

### 3.4. Shear Strength

**Figure 6:**
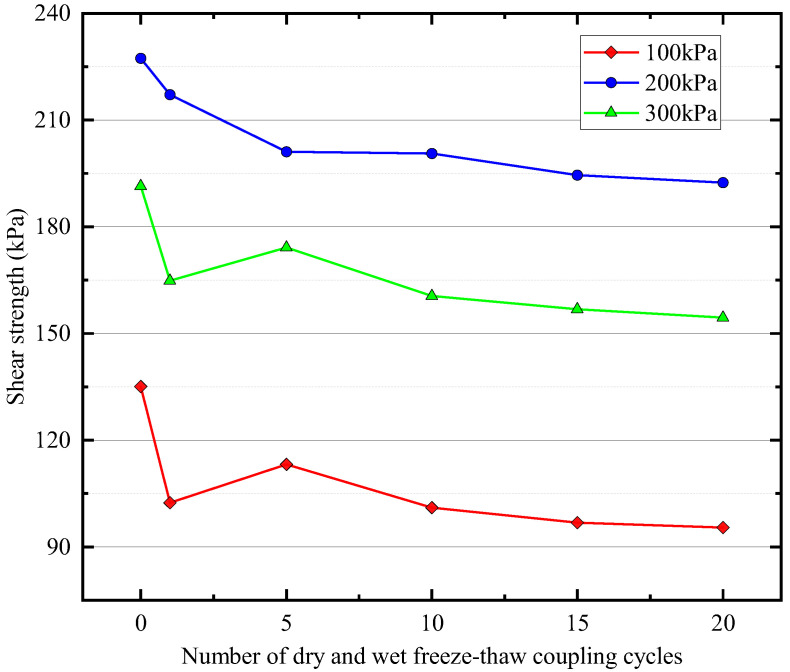
Shear strength variation curve.

Based on the calculated shear strength indices (c and φ), using the Mohr–Coulomb law and Equation (3), the shear strength of the soil was assessed, as shown in Figure 6. Under undrained unconsolidated (UU) conditions, the shear strength of the samples subjected to WD-FT coupled cycling was lower than that of the samples without such cycling, and this change was the most significant after the initial few cycles. The effect of WD-FT coupled cycling on the soil shear strength was stronger in the early stages. With an increase in the number of WD-FT coupled cycles, the shear strength of the samples fluctuated slightly before decreasing and eventually stabilizing between 15–20 cycles. The stabilized shear strength value was lower than that before WD-FT coupled cycling.

### 3.5. Changes in Microscopic Particle Structure of Loess

#### 3.5.1. Acquiring Microscopic Images

When conducting electron microscopy experiments, representative areas of fresh-cut surfaces were selected for imaging. Three typical areas were selected for each sample, and the best images were selected for the subsequent quantitative analysis.

#### 3.5.2. Microscopic Image Processing

**Figure 7:**
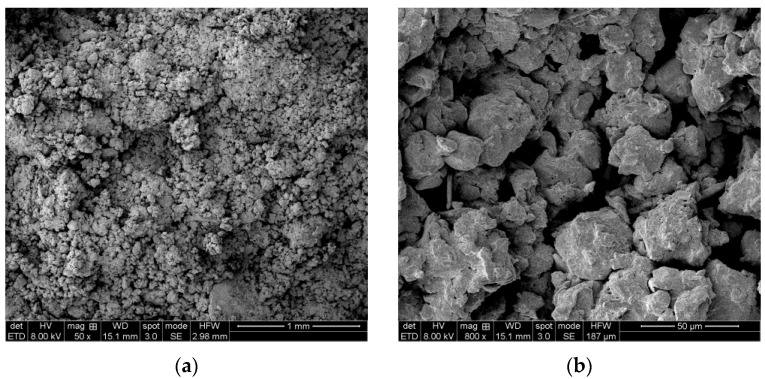

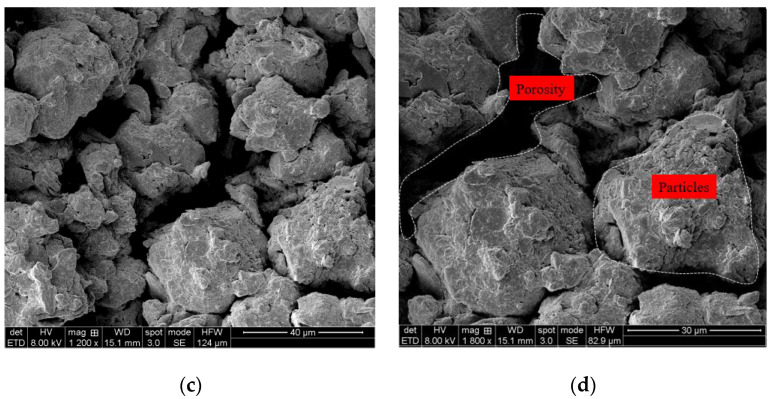
Scanning electron microscope image of partial loess specimen. Through the SEM images, the particles and pores of the soil can be seen. By comparison, it can be seen that the images with 1800× magnification are more obvious and easier to observe, so the images with 1800× magnification are uniformly chosen for analysis and processing. (**a**) Scanning electron microscope image at 50×; (**b**) Scanning electron microscope image at 800×; (**c**) Scanning electron microscope image at 1200×; (**d**) Scanning electron microscope image at 1800×.

The information on the structure of soil provided by the images scanned by an electron microscope is excessive. Image processing and analysis are widely applied to analyzing the microstructure characteristics of soil [27]. Figure 7 shows SEM images of loess samples with different magnifications after zero WD-FT cycles. Considering the images’ quality and the research object’s needs, images with a magnification of 1800 times were uniformly selected for analysis and processing.

### 3.6. Microscopic Experimental Results and Analysis

#### 3.6.1. Qualitative Analysis

**Figure 8:**
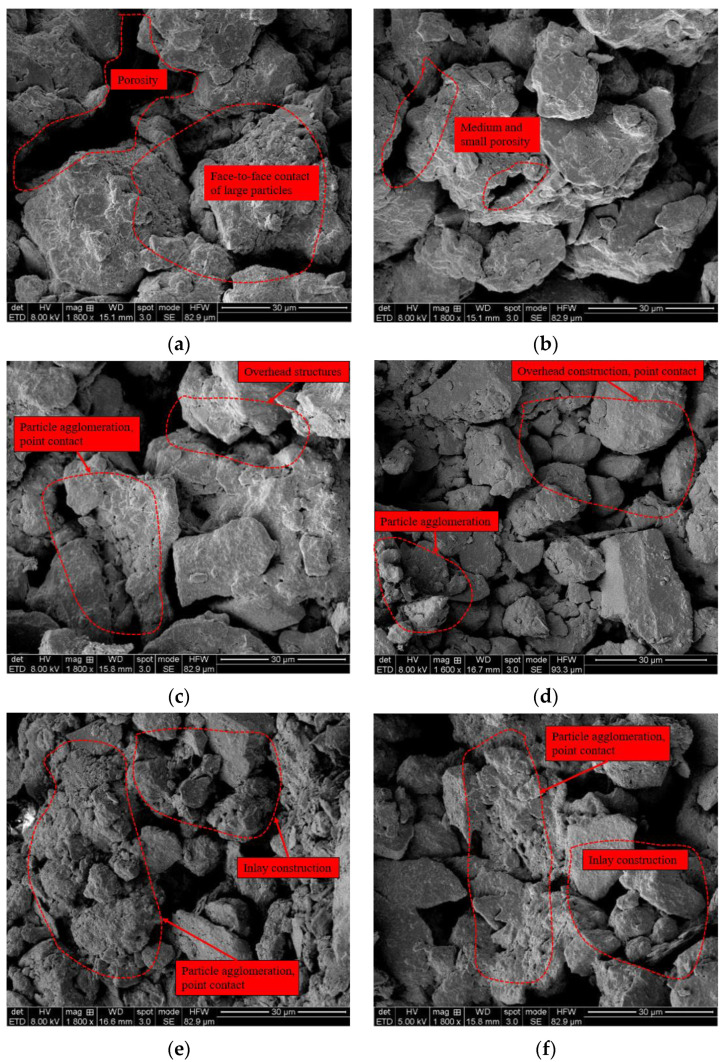
Scanning electron microscope images at different cycle times (**a**) Scanning electron microscope images at 0 WD-FT coupling cycles; (**b**) Scanning electron microscope images at 1 WD-FT coupling cycles; (**c**) Scanning electron microscope images at 5 WD-FT coupling cycles; (**d**) Scanning electron microscope images at 10 WD-FT coupling cycles; (**e**) Scanning electron microscope images at 15 WD-FT coupling cycles; (**f**) Scanning electron microscope images at 20 WD-FT coupling cycles.

By examining the SEM images (Figure 8), the morphology of the soil skeleton, the distribution of pores and particles, and the connection of contact zones were observed. From the microstructure image of the soil sample after different numbers of WD-FT coupled cycles, it can be seen that the arrangement of soil particles before the WD-FT cycling was a suspended-embedded pore type, and the interconnection between particles was a face-to-face contact. The soil skeleton particles were mostly large and medium-sized, and their particle size difference was relatively large. After experiencing several WD-FT coupled cycles, the fragmentation of large particles inside the soil became more pronounced. Large particles gradually broke and formed smaller particles during the WD-FT coupled cycling, destroying the original cohesive action inside the soil and causing particle redistribution. Some smaller particles agglomerated to form agglomerates and generate new cohesive forces, gradually filling large and small pores in the soil so that the pore size gradually decreased. Moreover, during this process, the number of face-to-face contacts in the skeleton connection gradually decreased, while those of point-to-point and point-to-face contacts gradually increased. From a microscopic perspective, Qi et al. [28] studied soil cohesion and internal friction angle. They attributed the soil cohesion variation to the change in the cohesive strength between soil particles, while the internal friction angle was affected by the roughness of the particles. With the increased number of WD-FT cycles, more cracks are generated in the soil, large particles become broken, and the cohesive action between particles is weakened, causing the soil cohesion to decrease with the number of WD-FT cycles.

As a shear strength parameter, the internal friction angle (φ) reflects the frictional force between soil particles resisting deformation during shear changes, mainly the sliding and biting frictions between soil particles. The distribution of fine particles in the soil after the WD-FT coupled cycling affects the internal friction angle. After experiencing WD or FT effects, soil particles are broken, and the generated fine particles are easily embedded into large pores, producing a “lubrication effect” on the sliding between particles. The friction between soil particles changes from biting to sliding, thus reducing the friction coefficient. Therefore, the internal friction angle of the soil decreases to a certain extent after the WD-FT coupled cycling.

#### 3.6.2. Quantitative Analysis

Quantitative analysis of soil microstructure mainly focuses on analyzing the size, distribution, shape, and orientation arrangement of soil particles (or unit cells) and pores between particles in the soil. The characteristic parameters of soil particles or pores are statistically processed to reveal the variation patterns of the microstructure after experiencing WD-FT coupled cycling. This study adopted Image-Pro Plus 6.0 software to perform binary processing (IPP, a digital image processing software, Version 6.0, Media Cybernetics, Inc., Rockville, MD, USA), image segmentation, and data statistics on the obtained electron microscope images. The SEM image only selects the information of one side of the particle and pore, but one SEM image contains a lot of information on each angle of the particle and pore, and the average value is used for quantitative analysis, which will replace the three-dimensional information, so the error is small. Since this user-friendly software allows selecting several parameters for measuring objects, according to users’ needs, we selected six basic parameters for studying the microstructure changes of loess based on SEM images: (i) the average diameter of particles and pores; (ii) average abundance; (iii) particle size fractal dimension or grain size dimensionality; (iv) particle roundness, i.e., short-to-long axis radius ratio; (v) directional probability entropy; and (vi) pore area ratio [29,30]. The equations for these microscopic parameters are given in Table 2.

#### 3.6.3. Image Information Extraction

**Figure 9:**
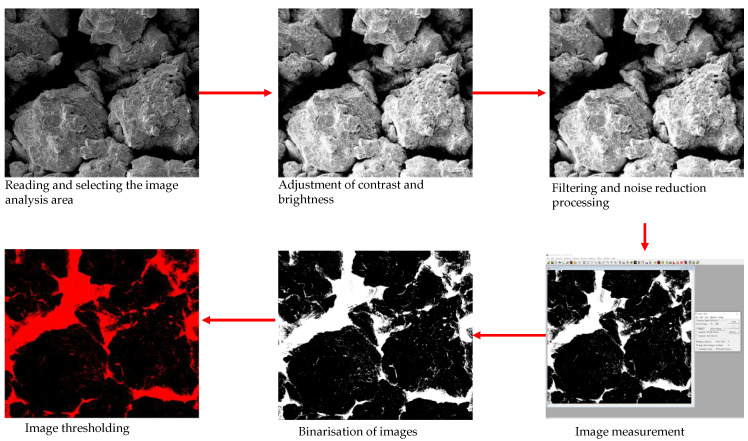
Schematic diagram of the image digitization process. (Example of loess under 0 WD-FT coupling cycles.).

The SEM images were digitized using Image-Pro Plus 6.0 software (its steps mainly include reading the loaded electron microscope image file; selecting the image analysis area; correcting the spatial scale; adjusting the image contrast; image filtering and noise re-reduction processing; selecting the image threshold, etc.), so as to extract soil pore-related parameters, including the average pore size, major axis, minor axis, actual perimeter, equivalent elliptical perimeter, and fractal dimension parameters. Then, each image is processed and removed in the same way to ensure minimal human variation. The general steps for digitizing images are shown in Figure 9. The calculated microscopic structural parameters are listed in Table 3.

**Figure 10:**
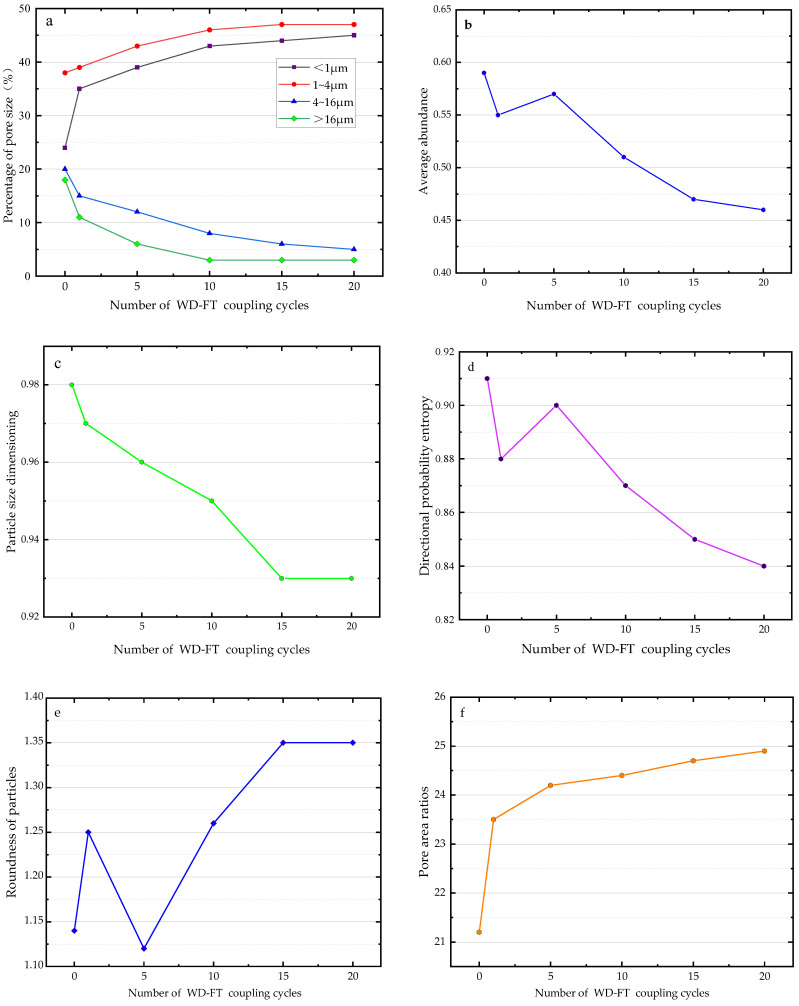
Relationship between the number of WD-FT coupling cycles and microstructural parameters (**a**) Variation in mean particle and pore diameters; (**b**) Average abundance; (**c**) Particle size dimensioning; (**d**) Directional probability entropy; (**e**) Roundness of particles; (**f**) Pore area ratio.

Based on the microstructure parameters obtained from SEM images, the dependencies between various parameters and the number of WD-FT coupled cycles were obtained and plotted in Figure 10. After a few WD-FT cycles, the number of soil particles with diameters of 4–16 µm and over 16 µm showed a decline, which was the most drastic after the first cycle, indicating that the large particles in the soil were decomposed after the first WD-FT cycle. With the increased number of cycles, the large particles continued to decompose, stabilizing after 15 cycles. At the same time, the soil particles were affected by the expansion force or wedge force of ice crystals formed by water, causing the large particles to collapse and decrease, reducing the number of large particles and producing more small and medium-sized ones, thus reducing the average particle diameter. The WD-FT coupled cycling also caused changes in the internal particles and pores of the soil, reducing their size (Figure 10a). After experiencing WD-FT coupled cycling, the average abundance showed a trend of slight fluctuation followed by a gradual decrease, indicating that the WD-FT coupled cycling promoted the roundness of the internal particles of the soil (Figure 10b). The particle size distribution dimension showed a trend of gradually decreasing and stabilizing after experiencing WD-FT coupled cycling. This indicates that the WD-FT coupled cycling arranged the soil’s internal particles more orderly (Figure 10c). The directional probability entropy of loess showed a similar effect to the average abundance after experiencing coupled cycling, with a trend of slight fluctuation followed by a gradual decrease and stabilization. Therefore, the WD-FT coupled cycling caused the internal particles of the soil to undergo redistribution. After redistribution, the internal structure of the soil became more orderly (Figure 10d). The particle roundness showed a trend of slight fluctuation followed by a gradual increase and stabilization after experiencing WD-FT coupled cycling. This indicates that the WD-FT coupled cycling promoted the roundness of the internal particles of the soil, making them more rounded (Figure 10e). After experiencing WD-FT coupled cycling, the pore area ratio showed a trend of first increasing and then stabilizing. This indicates that after experiencing WD-FT coupled cycling, the roundness of the internal particles of the soil was enhanced, and the number of points-to-point contacts between particles decreased, thus increasing the share of internal pores in the soil (Figure 10f).

### 3.7. Grey Correlation Results and Analysis

#### 3.7.1. Determination of the Analysis Sequence

This study analyzed the internal friction angle and cohesive force of loess under the coupled cycling effect of WD and FT cycles, which were macroscopically selected from the reference sequence and reference coefficient sequence and microscopically selected from the microstructure parameters obtained from SEM observations. The macro and micro parameters after different numbers of WD-FT cycles are listed in Table 4.

#### 3.7.2. Dimensionless Processing of Variables [31]

Given the different dimensions and units of various parameters in the reference and comparison sequences, it was necessary to normalize them before establishing their correlations. This involved initializing the raw data using the mean method.

#### 3.7.3. Calculating the Correlation Coefficient

After processing the approximate similarity Δ*_i_*(*k*), we calculated the difference between the comparison and reference coefficient sequences.

(5) $$\Delta_{i}(k)=\left|{\tilde{x}}_{i}(k)-{\tilde{x}}_{0}(k)\right|$$

For calculating the correlation coefficient of the processed data, the following formula was used:

(6) $$\xi i(k)=\frac{\min_{i}\min_{k}\Delta_{i}(k)+\rho \max_{i}\max_{k}\Delta_{i}(k)}{\Delta_{i}(k)+\rho \max_{i}\max_{k}\Delta_{i}(k)}$$

The processing results can be found in Table 5 and Table 6. Here parameters ζ1~ζ6 and ε1~ε6 represent the correlation coefficients between the selected microparameters and the internal friction angle and cohesive force, respectively.

#### 3.7.4. Correlation Assessment

The correlation coefficient is the degree of correlation between the reference and comparison sequences at each point, so it is a multiple value. Taking the average of these values yields a more accurate correlation degree.

(7) $$\gamma_{i}=\frac{1}{n}\sum_{k=1}^{n}\xi_{i}(k),k=1,2\cdots ,n$$

The gray correlation between various micro parameters and the permeability coefficient was calculated using Formula (7), as shown in Table 7. At ρ = 0.5 and $\;\gamma_{i}\geq 0.6$, the correlation between these factors was considered strong. At $\gamma_{i}<0.6$, the correlation was considered weak.

According to Table 7, the six microparameters selected in this article were ranked in order of their correlation with the internal friction angle from highest to lowest: grain size dimensionality > pore area ratio > particle roundness > average abundance > directional probability entropy > average diameter. This indicates that grain size dimensionality has the closest relationship with the macroscopic internal friction angle in the coupled WD-FT cyclic action. In contrast, the relationship between pore area ratio, particle roundness, average abundance, directional probability entropy, average diameter, and macroscopic internal friction angle decreased sequentially. This was mainly reflected in soil undergoing WD-FT coupled cyclic action, where the original distribution of particles in the soil was destroyed. In the process of particle redistribution, the arrangement of particles became more orderly, reducing the number of point-to-point contacts between particles. Moreover, the coupled cyclic action caused large particles in the soil to break, increasing the number of medium-sized pores and the pore area ratio. During this process, particle roundness gradually increased, reducing the number of point-to-point and point-to-face contacts between particles. The comprehensive changes in the above microparameters first increased the internal friction angle, gradually decreasing and stabilizing at the macroscopic level.

The correlation between porosity and cohesion was ranked from high to low as follows: porosity ratio > grain size fractal dimension > average diameter > directional probability entropy > particle roundness > average abundance. This indicates that porosity has the closest correlation with macroscopic cohesion in coupled WD-FT cyclic action, while correlations between grain size fractal dimension, average diameter, directional probability entropy, particle roundness, average abundance, and macroscopic cohesion gradually decreased. This was mainly reflected in the WD-FT coupled cycling, where the initial bonding between particles in the soil was destroyed, causing particle migration within the soil. During this process, some bonding materials (hydrophilic minerals) detached from the original soil particles, weakening the bonding force between the particles. The newly formed bonding force was smaller than the initial one, leading to a gradually decreasing and stabilizing trend of macroscopic cohesion.

## 4. Conclusions

This study used wetting-drying and freeze-thaw (WD-FT) coupled cycling tests, triaxial shear tests, and scanning electron microscopy (SEM) to analyze the changes in the shear strength index and micromechanisms of loess in the Yili region under WD-FT coupled cycling conditions experienced in the winter months. The gray correlation analysis method was used to analyze the correlation between the shear strength index and microstructure parameters of loess under WD-FT coupled cycling. The main findings of this study are as follows:

1. After undergoing WD-FT coupled cycling, the internal friction angle and cohesive force decreased to varying degrees. Both indices underwent the most drastic drop during the first WD-FT cycle; then, the degree of change gradually slowed, tending to stabilize with an increased number of cycles, eventually reaching a new stable state.
2. Under the unconsolidated undrained (UU) conditions, compared with the initial state of the sample, the shear strength of the sample subjected to WD-FT coupled cycling first dropped and then stabilized. The initial effect of WD-FT coupled cycling on the shear strength of the soil was the most significant. With the increased number of cycles, it stabilized below the initial one.
3. The SEM image analysis after WD-FT coupled cycling revealed numerous cracks generated in the soil/loess samples due to the cumulative effect of WD-FT cycles. Besides, large particles were broken, and the interparticle bonding was weakened, resulting in a gradual decrease in soil cohesion with increased cycles. The distribution of fine particles in the soil after WD-FT coupled cycling affected the internal friction angle, as soil particles broke and the generated fine particles easily became embedded into large pores, resulting in a “lubrication effect” on particle sliding and causing a certain degree of decrease in the internal friction angle of the soil.
4. The gray correlation analysis revealed that the grain size dimension closely correlated with the macroscopic internal friction angle during the coupled WD-FT cycling, while the correlation significance between the pore area ratio, particle roundness, average abundance, directional probability entropy, average diameter, and macroscopic internal friction angle decreased sequentially. The pore area ratio closely correlates with the macroscopic cohesion force during coupled WD-FT cycling. The correlation significance between grain size dimension, average diameter, directional probability entropy, particle roundness, average abundance, and macroscopic cohesion force decreased sequentially.
5. The wetting-drying cycle effect on soil strength was weaker than that of the freeze-thaw cycle. The first WD, FT, and WD-FT cycles produced the strongest effects on the soil’s mechanical and microstructural properties. From the standpoints of the internal friction angle, cohesion, and shear strength attenuation, the coupled WD-FT cycling effect had neither superposition nor synergetic pattern: it exceeded the effects of single (FT or WD) factors but was less than their sum. The follow-up study envisages a more detailed study of the FT and WD loading history effect in soil samples’ coupled FT and WD cycling.

## Figures and Tables

**Table 1:** Basic physical indicators of in-situ soils.

Natural Density	Natural Water Content	Maximum Dry Density	Saturated Water Content	Optimum Moisture Content
1.95 g/cm^3^	20.51%	1.86 g/cm^3^	24.32%	17.4%

**Table 2:** Each microstructure parameter and calculation method.

Parameters	Calculation Method	Remarks
Average diameter of soil particles as well as internal pores	D =$\sqrt{\frac{4S}{\pi }}$	None
Abundance (ratio of short axis to long axis)	C =$\frac{B}{L}$	Abundance is defined as the ratio of the short axis *B* to the long axis *L*.
Particle size fractal dimension	Dps = −$\underset{r\to 0}{\lim}\frac{\ln N\left(r\right)}{\ln\left(r\right)}$	Where: r is the particle size, N(r) is the number of particles exceeding the selected size.
Directional probability entropy	Hm = $\overset{n}{\underset{i=1}{\sum }}F_{i}\left(\alpha \right)\log_{n}F_{i}\left(\alpha \right)$	Where: *F_i_* (α) is the orientation frequency; Hm takes the value of 0~1.
Particle roundness	R = $\frac{P^{2}}{4\pi A}$	None
Pore area ratio	PAR = $\frac{A1}{A2}$	Where: PAR denotes pore area ratio (%), *A*1 denotes pore area, *A*2 denotes total image area.

**Table 3:** Summary of microstructural parameters.

Number of WD-FT Coupling Cycles	Average Particle and Pore Diameter	Abundance	Particle Size Dimensioning	Directional Probability Entropy	Roundness of Particles	Pore Area Ratio
0	2.58	0.59	0.98	0.91	1.14	21.2
1	2.40	0.55	0.97	0.88	1.25	23.5
5	2.34	0.57	0.96	0.9	1.12	24.2
10	2.19	0.51	0.95	0.87	1.26	24.4
15	1.96	0.47	0.93	0.85	1.35	24.7
20	1.95	0.46	0.93	0.84	1.35	24.9

**Table 4:** Table of macro and micro parameters of loess with different numbers of WD-FT coupling cycles.

Number of WD-FT Coupling Cycles		0	1	5	10	15	20
Reference Department	Angle of internal friction	23.52	23.11	23.61	23.01	22.18	22.15
	Cohesive force	80.45	78.21	81.94	78.66	74.31	73.11
Comparative Department	Average diameter	2.58	2.40	2.34	2.19	1.96	1.95
	Average abundance	0.59	0.55	0.57	0.51	0.47	0.46
	Particle size fractional dimension	0.98	0.97	0.96	0.95	0.93	0.93
	directional probability entropy	0.91	0.88	0.9	0.87	0.85	0.84
	Average diameter	2.58	2.40	2.34	2.19	1.96	1.95
	Average Abundance	0.59	0.55	0.57	0.51	0.47	0.46

**Table 5:** Correlation coefficients of macro and micro parameters (angle of internal friction).

ζ1	ζ2	ζ3	ζ4	ζ5	ζ6
0.56	0.34	0.78	0.47	0.87	0.41
0.39	0.69	0.39	0.33	0.25	0.26
0.85	0.36	0.85	0.55	0.40	0.34
0.60	0.43	0.56	0.41	0.62	0.42
0.43	0.44	0.38	0.47	0.52	0.45
0.54	0.75	0.48	0.66	0.79	0.52

**Table 6:** Correlation coefficients of macro and micro parameters (cohesion).

ε1	ε2	ε3	ε4	ε5	ε6
0.46	0.38	0.71	0.42	0.53	0.47
0.41	0.51	0.42	0.38	0.35	0.31
0.75	0.42	0.87	0.60	0.51	0.42
0.64	0.52	0.62	0.46	0.76	0.50
0.51	0.35	0.55	0.58	0.64	0.39
0.43	0.81	0.48	0.71	0.81	0.53

**Table 7:** Correlation coefficients of microscopic parameters and strength index.

Compare Series	Gray Correlation	
	Angle of Internal Friction	Cohesive Force
Average diameter	0.43	0.73
Average abundance	0.59	0.61
Directional probability entropy	0.56	0.65
Particle roundness	0.61	0.64
Pore area ratio	0.64	0.66
Particle size fractional dimension	0.69	0.72

## Data Availability

The data used to support the findings of this study are included within the manuscript.
